# Synthesis, Identification, and Biological Study for Some Complexes of Azo Dye Having Theophylline

**DOI:** 10.1155/2021/9943763

**Published:** 2021-07-21

**Authors:** Hasan Mohammed

**Affiliations:** Chemistry Department, Science College, University of Al-Qadisiyah, Al Diwaniyah, Iraq

## Abstract

This article includes the synthesis of heterocyclic azo dye of theophylline by coupling diazonium salt of 4-chloroaniline with theophylline which is, namely, 8-(1-(4-chlorophenyl)azo)theophylline (CPAT). The complexes of cobalt and nickel were prepared by reacting their ions with CPAT ligand in ethanol under 1 : 2 ratio metal-ligand. The CPAT ligand and its complexes were characterized by elemental analysis, infrared spectrometry, electronic absorption spectroscopy, molar conductivity, and magnetic moment. The cobalt and nickel complexes show octahedral geometry having general formula [M(CPAT)_2_Cl_2_]. This article addresses the properties of CPAT dye such as photochromic properties. The CPAT dye exhibited obvious and desired changes under irradiation with visible light (405 nm), high sensitive for pH changes which refer to its ability to be analysis indicator. CPAT dye exhibited solvatochromic properties presenting red shift with polar solvent. The CPAT and its complexes show interesting antibiological activities towards *Staph. aureus* and *E. coli* bacteria and *Aspergillus* fungi.

## 1. Introduction

The azo function (-N=N-) in the ligands appears intense color within the visible area and is sensitive for the changes in the acidity (pH) that leads to the use of the ligands of this function as colorant for the tissue and indicators in analytical chemistry [1–4]. Azo dyes exhibit stereoisomerism under the light. The trans isomer is stable which converts to cis isomer under the light, when the process is perfectly reversible calling photochromic [5]. When this process is accompanied by a significant change in the dipole moment, that qualifies the material to be a candidate for high optic data storage [6]. The azo dye complexes such as azo quinoline dye and its complexes and rhenium complex based on the azo dye of iminopyridine exhibited significant nonlinear optical properties, and these properties play an important role in optical data storage and telecommunications [7–9].

The azo dyes have been received big attention as reagents to extract and determine the trace amount of metal ions in different samples [10–12]. Azo dye complexes were studied extensively due to their interesting properties and their applications such as catalysts, antimicrobials, colorants, corrosion inhibitors, and anticancer [13–15]. Azo complexes such as sulfamethoxazole azo dyes [16] and pyrazole azo complexes [17] exhibited interesting activities towards *tuberculosis*.

The complexes of the azo dye of 2,6-diaminopyridine showed a significant efficiency against *Escherichia coli* and *Staphylococcus*. On the other hand, these complexes exhibited high inhibitory activity against breast cancer (CMCF7 cell line) with high-interest IC_50_ which was 3.5–7.0 *μ*gmL^−1^ [10]. Azo dyes are used for the treatment of viral infections, for example, in the presence of Direct Red 23 (azo dye), the ability of the virus to bind to the CD4 glycoprotein inhibits. Bismarck brown dye is used for the qualitative detection of Sb^+3^ ion. Azo dyes and azo dye complexes are used as photosensitizers [18] such as ruthenium complexes with the azo dye of quinolone exhibiting anticancer potential because they show significant activity in photodynamic therapy at long wavelength [19]. Azo dyes and azo dye complexes are used as photosensitizers in two-photon photodynamic therapy for the treatment of cancer because they exhibit low toxicity in dark conditions and a high yield for reactive oxygen species and are suitable for two-photon absorption [20, 21].

The high pi acidity of N-heterocycles of azo dyes gives stability for the range of oxidation states of metal ions. Large amounts of azo dyes are added to food products to totalize the appearance and nutritional properties [22, 23]. Azo dyes and their complexes such as azo dye chromium (III) especially with acid dye exhibited large uses as dying in writing ink, toners for photocopiers, and dying for leather and hair [24]. Due to those applications of the azo dyes in industry, medicine, and spectroscopic analysis, we are interested in the preparation of azo dye of theophylline and its complexes with positive divalent ions of cobalt and nickel and study their spectroscopic and biological properties.

## 2. Experimental

### 2.1. Material and Methods

All reagents and solvents are from Sigma Aldrich except for theophylline which is supplied from CDH Company. Melting points of azo dye of theophylline and its complexes were recorded by electrothermal apparatus. Elemental analysis of CHN was done on Vario EIIII CHN analyser. Infrared spectra of CPAT ligand and the complexes were done on a Perkin-Elmer, using KBr disks. ^1^H-NMR spectrum of CPAT dye was done in DMSO-d6 by Bruker Avance II spectrometer. LC-MS spectra of our compounds were carried out on LCMS 2010, Shimadzu mass analyser. The measurements of molar conductivity for synthesized complexes of CPAT dye were achieved on an ELICO CM-180 conductivity bridge in DMSO (10^−3^ M). The measurements of magnetic moment were done at room temperature using MSB balance. Electronic spectra were done by Shimadzu-1800.

Synthesis of azo dye of theophylline (CPAT) : the solution of 4-chloroaniline (1.0 g, 7.8 mmole) was mixed with 6 mL HCl (12N), and then the acidic aqueous solution of 4-chloroaniline was cooled at 0–5°C. 10 mL of the cold aqueous solution (0.54 g, 7.8 mmole) of NaNO_2_ was added to the acidic solution of 4-chloroaniline to form diazotization salt of 4-chloroaniline. Theophylline (1.4 g, 7.8 mole) was dissolved in 40 mL of aqueous solution of sodium hydroxide (1.6 g). The diazotization salt of 4-chloroaniline was mixed with cold basic solution of theophylline. The solution was left overnight, the solution was filtrated, and the formed azo dye was dark yellow powder, yield: 60%; IR cm^−1^: 3371 (N–H), 3105 (CH aromatic), 2919 (CH aliphatic), 1705 (C=O), 1641 (C=N), 1556 (C=C), and 1444 (N=N); elemental analysis % (C.H.N) of C_13_H_11_N_6_O_2_Cl; C = 48.56 (cal. 48.99), H = 3.35 (cal. 3.48), and N = 26.31 (cal. 26.37); and m.p. 270°C.

The coordination compounds of cobalt and nickel (divalent) for CPAT dye were synthesized by reacting hot aqueous solution (10 mL) having 0.3 mmole of these salts in each case with 15 mL hot ethanol having (0.2222 g, 0.3 mmole) of CPAT dye. The mixture of reaction was stirred for one hour, and then the solution was filtrated: Co(II) complex: 80% yield, brown powder; IR cm^−1^: 3444 (N–H), 3064 (CH aromatic), 2900 (CH aliphatic), 1653 (C=O), 1606 (C=N), 1531 (C=C), and 1417 (N=N); elemental analysis % (C.H.N) of C_26_H_22_N_12_O_4_Cl_4_Co; C = 40.58 (cal. 40.70), H = 2.75 (cal. 2.89), and N = 21.83 (cal. 21.91); and m.p. more 375°C, as well as Ni(II) complex: 75% yield, yellowish green powder; IR cm^−1^: 3421 (N-H), 3100 (CH aromatic), 2900 (CH aliphatic), 1676 (C=O), 1616 (C=N), 1531 (C=C), and 1417 (N=N); elemental analysis % (C.H.N) of C_26_H_22_N_12_O_4_Cl_4_Ni; C = 40.53 (cal. 40.71), H = 2.76 (cal. 2.89), and N = 21.73 (cal. 21.91); and m.p. more 375°C.

Results of bioactivity against Gram-positive and Gram-negative bacteria and fungi were performed against each of the epidermal *Staphylococcus*, *E. coli*, and *Aspergillus* fungi by the method of propagation on Petri dishes, using a Muller-Hinton medium for all compounds at concentrations of 100 and 50 mg/mL. The damping zone diameter was determined for each trial by taking the mean for repeated three times for each trial.

## 3. Results and Discussion

**Scheme 1:**
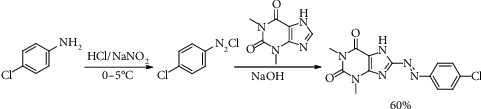
Preparation steps of CPAT azo dye of theophylline.

In this study, azo dye of 8-(1-(4-chlorophenyl)azo)theophylline (CPAT) was synthesized by diazotization of 4-chloroaniline, then coupling the formed diazotization salt with theophylline in basic aqueous solution to form the dye as in Scheme 1.

**Figure 1:**
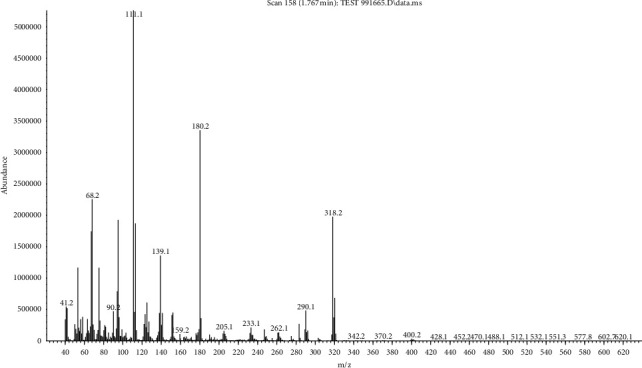
Mass spectrum of CPAT dye.

The mass spectrum of CPAT dye is depicted in Figure 1. The CPAT dye exhibited a peak *m*/*z* = 318 due to the mother fragment which is in agreement with expected molecular mass of the CPAT dye, and it is perfectly in agreement with C.H.N analysis of CPAT dye.

**Figure 2:**
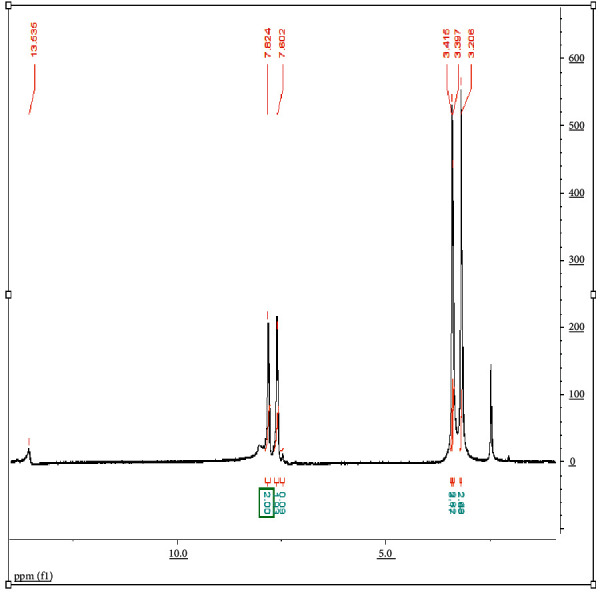
^1^H-NMR spectrum of CPAT dye in DMSO-d6 at 298 K.

The CPAT dye was also characterized by ^1^H-NMR spectroscopy in DMSO-*d*6 solvent. The ^1^H-NMR spectrum of CPAT (Figure 2) exhibited five signals besides the signals of solvent and water. The first signal is resonating at 13.5 ppm and attributed to N-H proton. The second and third signals are the two doublet peaks attributed to the four protons (each signal is due to two protons) of the aromatic ring which are resonating at 7.8 ppm and 7.6 ppm. The remaining fourth and fifth signals are attributed to the two methyl groups of theophylline that resonate at 3.4 and 3.2 ppm.

**Figure 3:**
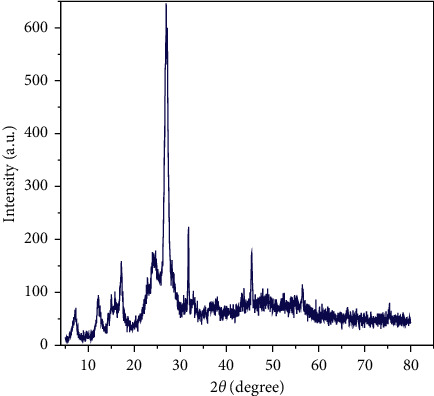
XRD spectrum of CPAT dye.

**Figure 4:**
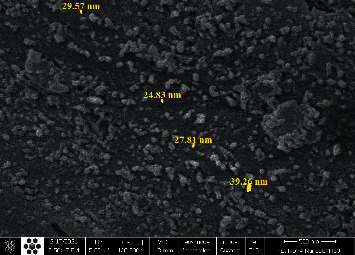
FESEM image of CPAT dye.

The XRD spectrum of CPAT dye is depicted in Figure 3 which was recorded in the 2*θ* range of 5–80. The position of signals (2*θ*) and full width at half maximum (FWHM) of signals were detected by X'Pert HighScore program. The crystalline size average of CPAT dye is calculated by Scherer's equation (*D* = 0.94*λ*/*β*Cos*θ*), the *D* represents the particle size of the crystal gain, *θ* represents Bragg diffraction angle, *λ* represents the X-ray wavelength which is 1.5406 Å, and *β* represents the integral peak width. The crystalline size average of CPAT dye is equal to 50 nm. The XRD exhibited sharp seven signals which indicate a well-defined crystalline structure for CPAT dye. The morphology of CPAT dye was investigated by field emission scanning electron microscopy (FESEM). The FESEM image of CPAT dye (Figure 4) shows a uniform distribution of particle size. The shape of CPAT dye particles is a beard, and the average particle size of CPAT was found to be between 24.8 and 39.26 nm, indicating that the particle size of CPAT dye is within the nanomaterial scale.

**Figure 5:**
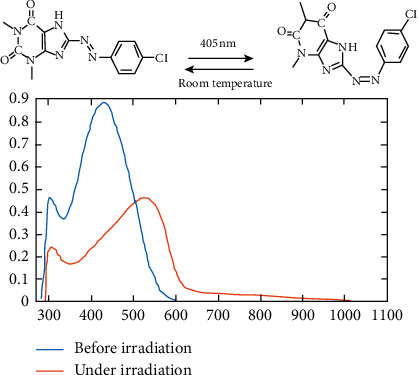
UV-visible absorption spectra of CPAT before (blue line) and under irradiation (red line) at 405 nm in acetone.

Photochromic properties of azo dyes have large application such as high optic data storage devices and photochromic inks [25, 26]. The visible light irradiation for CPAT dye at 405 nm exhibited high conversion from trans (*E*) to cis (*Z*) geometry, and these changes were monitored by UV-visible absorption spectroscopy (Figure 5). It seems that *π*–*π∗* transition overlaps with *n*-*π∗* to form large band at 424 nm in acetone which leads to decrease energy of *π*-*π∗* transition and increasing chance of converting of trans to cis isomer [9, 27]. When the dye coordinates to the metal within the complex structures, the photochromic properties will be lost [9]. In some dye complexes, the dye does not bond to the metal; therefore, it still exhibits photochromic properties. In our case, the complexes did not show photoisomerization behavior which means that the CPAT dye is bonded to the metal within complex structure. It is nice to point that the cis isomer backed totally at room temperature to trans isomer which means that the chromic behavior is intrinsic [27–32].

### 3.1. Solvatochromic Studies of CPAT Dye

**Figure 6:**
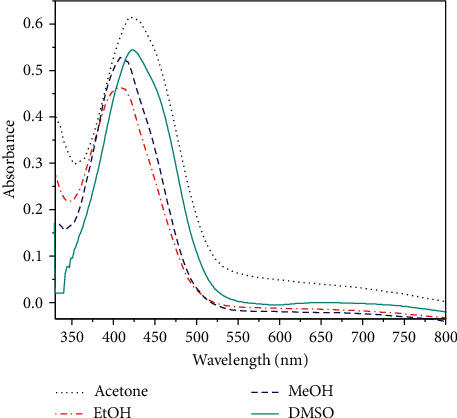
Electronic absorption spectra of 10^−4^ M CPAT dye in different solvents at ambient temperature.

Solvatochromism concept refers to change in the color by the interaction of the solvent with a solute which produces changes in the size and the shape of the UV-Vis spectra. When the solvent is compatible and suitable for the dye (solute), this gives precision to the UV-Vis spectroscopy method. The absorption spectra of 10^−4^ M CPAT dye (Figure 6) have been recorded in four solvents having different polarities at ambient temperature. The CPAT dye in different solvents proved red shift with hyperchromic effect under decreasing the polarity from methanol, ethanol, and DMSO to acetone which let to use it as solvatochromic probe [2, 33, 34]. The CPAT dye exhibited clear absorbance shifts (hypochromic or hyperchromic) which can project a good idea of the interaction pattern between the solvent and dye (solute).

### 3.2. CPAT Dye and Its Complexes of Co(II) and Ni(II)

**Scheme 2:**
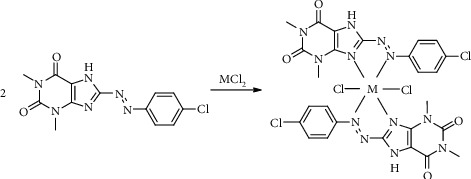
Preparation of CPAT complexes for Co(II) and Ni(II).

**Figure 7:**
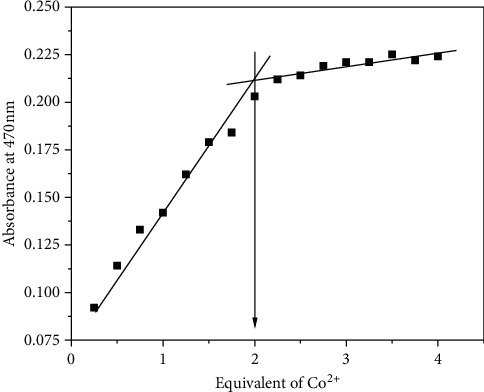
The visible absorption titration of 0.2 mM.

The complexes of CPAT with Co(II) and Ni(II) were synthesized in ratio of M : L (1 : 2) (Scheme 2). This ratio was confirmed by elemental analysis (C.H.N), molar conductivity, infrared spectrometry, and the visible titration of 2 equivalent dye ligand with 1 equivalent of cobalt chloride salt (Figure 7).

### 3.3. CPAT with 0.2 mM Cobalt Chloride in DMSO at Room Temperature

**Figure 8:**
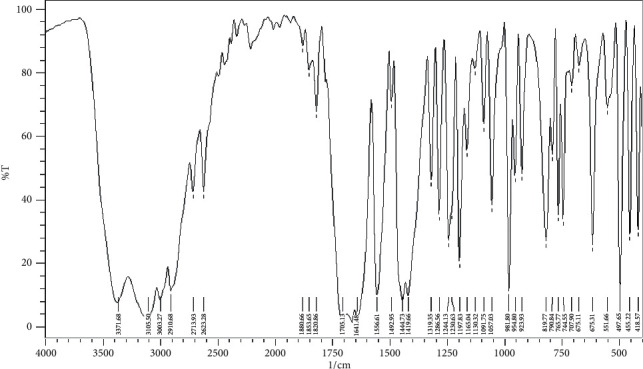
Infrared spectrum of CPAT as KBr disk.

**Figure 9:**
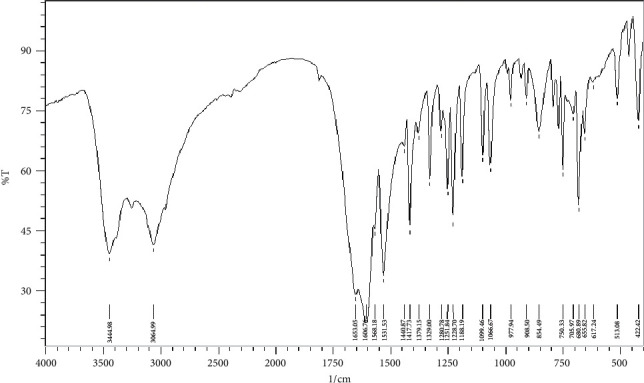
Infrared spectrum of cobalt complex as KBr disk.

**Figure 10:**
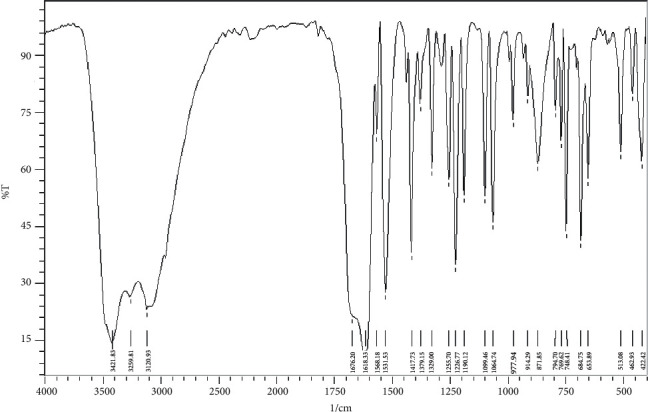
Infrared spectrum of nickel complex as KBr disk.

The infrared spectra of CPAT dye and the Co(II) and Ni(II) complexes are depicted in Figures 8–10. These spectra show important characteristic peaks including stretching vibrations of NH, aromatic CH, aliphatic CH, C=O, C=N, and N=N. We noticed that the frequencies of C=N and N=N groups show red shift in the complexes of cobalt and nickel compared to those in the spectrum of the free CPAT ligand. The C=N group exhibited red shift of 25 cm^−1^ in the spectra of cobalt and nickel complexes. The azo group (N=N) exhibits red shift of 27 cm^−1^ in both complexes in agreement with the literature related to azo dye complexes [14, 35].

**Figure 11:**
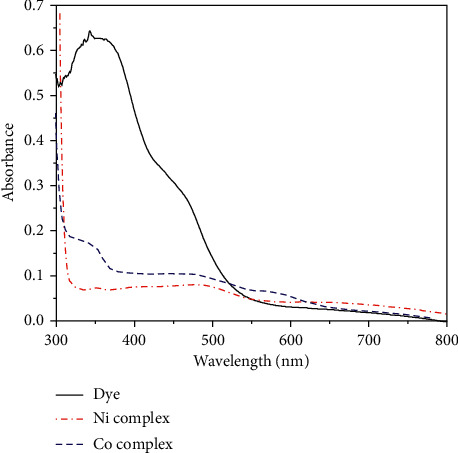
Electronic absorption spectra of CPAT, cobalt, and nickel complexes in DMSO at ambient temperature.

Electronic spectra of CPAT dye and the complexes were recorded in DMSO at room temperature and are depicted in Figure 11. The CPAT dye showed absorption bands at 280 nm and 350 nm due to *π*-*π*^∗^ transition and at 450 nm due to *n*-*π*^∗^ transition. Nickel (II) complex of CPAT dye exhibited absorption bands at 343 nm due to *π*-*π*^∗^ transition, 471 nm due to ^3^A_2g_(F) to ^3^T_2g_(F) transition, 620 nm due to ^3^A_2g_(F) to ^3^T_1g_(F) transition, and 717 nm due to ^3^A_2g_ (F) to ^3^T_2g_(P) transition, with a magnetic moment equal to 3.21 B M in agreement with octahedral nickel complexes [36]. Cobalt(II) complex of CPAT dye showed absorption bands at 350 nm due to *π*-*π*^∗^ transition, 470 nm due to ^4^T_1g_(F) to ^4^T_2_g(P) transition, 580 nm due to ^4^T_1g_(F) to ^4^A_2g_(F) transition, and 705 nm due to ^4^T_1g_(F) to ^4^T_2g_(F) transition with magnetic moment equal to 5.01 B.M. in agreement with octahedral cobalt complexes [35, 36]. The molar conductance of the synthesized cobalt and nickel complexes (10^−3^ M) were 15 and 20 S cm^2^ mol^−1^, respectively, in DMSO solvent which indicated that both these complexes are nonionic.

### 3.4. Biological Activity of CPAT and Its Complexes

**Figure 12:**
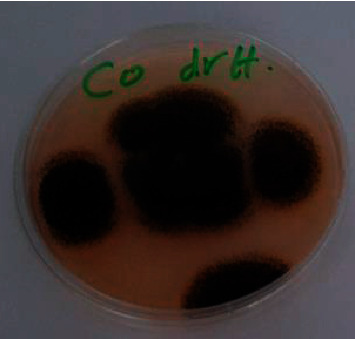
Inhibition zones of cobalt complex against *Aspergillus* fungi.

Antimicrobials are critical for reducing the global burden of infectious diseases. Antimicrobial resistance is spreading around the world, reducing the efficacy of many antibiotics, particularly in immunocompromised patients. Drug resistance, especially by bacteria and fungi, is a major concern for public health and scientific communities around the world. The antimicrobial activity of CPAT dye and its complexes are performed against *E. coli* and *Staph. aureus* bacteria and *Aspergillus* fungi by diffusion technique. The antimicrobial data, as shown in Table 1, indicated that CPAT dye and its synthesized complexes exhibited significant biological activities against these organisms where the synthesized compounds exhibited inhibition zones from 2.5 to 3.5 cm against *E. coli* bacteria and 1 to 2.5 cm against *Staph. aureus* bacteria. The compounds exhibited 3–4 cm inhibition zones against *Aspergillus* fungi. The cobalt complex showed the highest activity against *Aspergillus* fungi (Figure 12). The complexes exhibit important biological activity comparing to CPAT dye and the standard drug (ciprofloxacin and fluconazole) that is possible due to the chelation in complexes which leads to a decrease in the polarity of the metal ions. This behavior increases the lipophilicity of the metals; therefore, the complexes permeate the lipid membranes of microorganisms and prevent their growth.

## 4. Conclusion

Novel azo dye of theophylline, namely, 8-(1-(4-chlorophenyl)azo)theophylline (CPAT), was prepared by coupling diazoinium salt of 4-chloroaniline with theophylline. The CPAT ligand is a good photochromic material, and it can be considered a candidate as a material for high optic data storage devices. The CPAT dye showed azo-hydrazone tautomers. The nitrogen of the azo group and nitrogen of the C=N group of CPAT dye are coordinated to the metal ion in complexes of cobalt and nickel which means that CPAT dye is a bidentate ligand. Both the cobalt(II) and nickel(II) complexes of CPAT dye exhibited octahedral geometries. In comparison to traditional antibiotics, the synthesized compounds in our study showed promising effectiveness against *Staph. aureus* and *E. coli* bacteria and *Aspergillus* fungi.

## Figures and Tables

**Table 1:** Inhibition zone (cm) of synthesized compounds against *E. coli* and *Staph. aureus* bacteria and *Aspergillus* fungi.

Compound	*E. coli*	*Staph. aureus*	*Aspergillus*
CPAT dye	3.5	1	3
Co complex	2.5	2	4
Ni complex	3	2.5	3
Ciprofloxacin	1.5	2.5	
Fluconazole			4.5

## Data Availability

The data used to support the findings of this study are available from the corresponding author upon request.
